# Risk factors and incidence of suction loss during small incision lenticule extraction (SMILE) in 8493 eyes

**DOI:** 10.1186/s12886-020-01680-x

**Published:** 2020-10-16

**Authors:** Tian-Ze Huang, Ling Shen, Xiao-Ning Yu, Hong-Ying Jin

**Affiliations:** 1 Chinese Academy of Medical Sciences, Peking Union Medical College, Beijing, China; 2 Eye Center, Second Affiliated Hospital, School of Medicine, Zhejiang University, No. 88 Jiefang Road, Hangzhou, China

**Keywords:** Suction loss, Risk factors, Small incision lenticule extraction, Refractive surgery

## Abstract

**Background:**

To report the incidence and risk factors of suction loss during small incision lenticule extraction (SMILE).

**Methods:**

This retrospective comparative case control study included 8493 eyes of 4261 patients. Patients underwent SMILE surgery between January 2014 and September 2019 were included. Videos of suction loss were reviewed, and the direct causes of suction loss were noted. An independent samples t-test was used for comparisons between the suction loss group and the control group. A binary logistic regression model was used to determine the possible significant risk factors that might increase the likelihood of suction loss during SMILE surgery.

**Results:**

Suction loss occurred in 31 (0.37%) eyes of 30 patients; 23 (74.2%) cases occurred in the right eye (the first operative eye) and 8 (25.8%) cases occurred in the left eye. Among the 30 patients, 23 (76.7%) were male and 7 (23.3%) were female. The incidence in the six consecutive years were 0, 2.13, 0.34, 0.24, 0.22, and 0.25%. Head and eye movements during surgery caused suction loss in 16 (51.6%) and 15 (48.4%) eyes, respectively. Comparison between the suction loss group and the control group showed that the first operative eye and male sex are at a significantly high risk for suction loss (*p* < 0.05).

**Conclusions:**

The risk factors of suction loss were first operative eye and male sex. Head and eye movements due to patient anxiety are the most common direct causes of suction loss. Surgeon’s experience may help to reduce the incidence of suction loss. Preoperative education and better communication during surgery needs to be emphasized.

**Trial registration:**

Retrospectively registered. ChiCTR-ORC-17011040. Registered 1 April 2017. Name of registry: The observation of clinical results after corneal refractive surgery. Data of enrolment of the first participant to the trial: 1 January 2014.

## Background

Small incision lenticule extraction (SMILE) is a flapless, minimally invasive refractive surgery that is currently gaining acceptance and popularity worldwide as it has potentially fewer complications than laser-assisted in situ keratomileusis (LASIK) and femtosecond laser-assisted LASIK (FS-LASIK). The efficacy, safety, predictability, and stability of SMILE have been supported by many clinical studies [1, 2]. However, intraoperative and postoperative complications can still be observed [3–7]. The possible complications of SMILE surgery include suction loss, difficulty or inability to extract the lenticule, and abrasion or minor tears in the small incision [8, 9]. Suction loss exerts potential negative effects on postoperative visual acuity, with an incidence ranging from 0.17 to 5.06% [10, 11].

Suction loss during SMILE procedure can result from several events, such as sudden eye rotation and eyelid squeezing [4]. When suction loss occurs, a laser machine will immediately shift into the repair mode [4]. According to the instructions given by the VisuMax device, surgery may be completed either by continuing with SMILE or by converting to LASIK. Reinstein has described five categories of suction loss according to laser cutting progress and provided recommendations for their management [4].

This paper reports on the risk factors and incidence of suction loss during SMILE surgery in a large population. We reviewed the videos of all suction loss cases in the SMILE procedures performed by one surgeon, and identified the causes of suction loss and recorded the management employed for each case.

## Subjects and methods

This is a retrospective comparative case control study of all myopic SMILE procedures performed consecutively between January 2014 and September 2019 by one surgeon (JHY) using a VisuMax Femtosecond Laser at the Eye Center of the Second Affiliated Hospital, School of Medicine, Zhejiang University. A total of 4261 patients were included. This research followed the tenets of the Declaration of Helsinki, and informed consent was obtained from the subjects. This study was approved by the institutional review board of the abovementioned hospital (No: 2017–017).

Surgical technique.

A VisuMax Femtosecond Laser System (Carl Zeiss Meditec AG, Jena, Germany) was used for surgical refractive correction in all patients, with a repetition rate of 500 kHz and a pulse energy of 155 nJ. The surgical procedure was detailed in our previous reports [1, 12]. In brief, a small patient interface cone (size S) was used in all patients, and prior to suction initiation, the patients were instructed to fixate on the green target light. Four cleavage planes were created, namely, the posterior surface of the refractive lenticule (spiral in), the lenticule border, the anterior surface of the refractive lenticule (spiral out), and the small incision. Cap thickness was adjusted for each patient to ensure that the residual stromal bed was maintained at more than 270 mm. The minimum lenticule side-cut thickness was set at 10 μm. A single side-cut incision (width: 2 to 4 mm) was made at 120°. Usually, the first operative eye is the right eye. When the laser treatment was completed for both eyes, the refractive lenticule was dissected through the side cut and removed manually using forceps.

Videos of all cases of suction loss were reviewed, and the surgical stage in which suction loss occurred and the different types of movements of eye and head were summarized. The management strategies employed to address suction loss was also identified. A control group was assembled by randomly selecting eyes from patients who had uneventful SMILE surgery.

### Statistical analysis

Statistical analysis was performed using the SPSS software (version 18.0, SPSS, Inc.). An independent samples t-test was used for comparisons between the suction loss group and the control group. A binary logistic regression model was used to determine the possible significant risk factors that might increase the likelihood of suction loss during SMILE surgery. The analyzed potential risk factors included age, sex, eye laterality, central corneal thickness (CCT), flat K readings, steep K readings, average K reading, sphere, astigmatism, residual stromal thickness, and optical zone. A *P* value of less than 0.05 was considered statistically significant.

## Results

Out of 8493 eyes of 4261 consecutive patients treated during the study period, 31 (0.37%) eyes of 30 patients had experienced suction loss. Among all the suction loss events, 23 (74.2%) occurred in the right eye and 8 (25.8%) occurred in the left eye. One patient experienced suction loss in both eyes. Among the 30 patients, 23 (76.7%) patients were male and 7 (23.3%) were female. The characteristics of the eyes in the suction loss and control groups (no suction loss) are described in Table 1. The binary logistic regression showed that the first operative eye and male sex were at a significantly higher risk of developing suction loss (*p* < 0.05). No other characteristic was a significant risk factor.

**Table 1:** Demographic data and patients’ information (mean ± SD)

Parameter	Suction loss group (*n* = 31)	Control group (*n* = 93)	*P*
OD/OS	23/7	41/52	.003*
Sex (M/F)	24/7	46/47	.005*
Age (years)	24.32 ± 7.23 (18 to 38)	24.03 ± 6.04 (18 to 45)	.826
IOP (mmHg)	15.55 ± 2.05 (12 to 20.50)	15.66 ± 2.32 (10.50 to 21)	.804
CCT (um)	551 .29 ± 25.44 (518 to 619)	547.45 ± 28.93 (496 to 621)	.551
Mean corneal power (D)	42.80 ± 1.22 (40.80 to 46.10)	43.11 ± 1.27 (40.85 to 45.85)	.238
Flat power (D)	42.27 ± 1.18 (40.40 to 45.20)	42.69 ± 1.31 (40.10 ± 46.10)	.112
Steep power (D)	43.35 ± 1.33 (41.20 to 47.00)	43.54 ± 1.41 (40.70 to 46.30)	.517
SE (D)	−4.57 ± 1.93 (−1.75 to −9.50)	−4.58 ± 1.75 (−2.00 to −8.50)	.981
Sphere (D)	−4.27 ± 1.65 (−1.75 to −8.25)	−4.23 ± 1.76 (− 1.50 to − 8.50)	.893
Cylinder (D)	−0.59 ± 0.56 (0 to −2.50)	−0.70 ± 0.59 (0 to − 2.50)	.352
Cap thickness (μm)	124.03 ± 6.88 (110 to 140)	123.76 ± 6.74 (110 to 140)	.849
Cap diameter (μm)	7.48 ± 0.07 (7.20 to 7.50)	7.49 ± 0.05 (7.1 to 7.60)	.329
Lenticule thickness (μm)	92.81 ± 25.77 (61 to 145)	94.55 ± 24.01 (60 to 148)	.732
Lenticule diameter (mm)	6.48 ± 0.07 (6.20 to 6.60)	6.49 ± 0.05 (6.10 to 6.50)	.329
RST	334.13 ± 37.18 (278 to 415)	329.14 ± 30.36 (278 to 418)	.444

*SD* Standard deviation, *OD* Right eye, *OS* Left eye, *M* Male patient, *F* Female patient, *IOP* Intraocular ocular pressure, *CCT* Central corneal thickness, *D* Diopter, *SE* Spherical equivalent, *RST* Residual stromal thickness, *control group* No suction loss. *p < 0.05

**Fig. 1:**
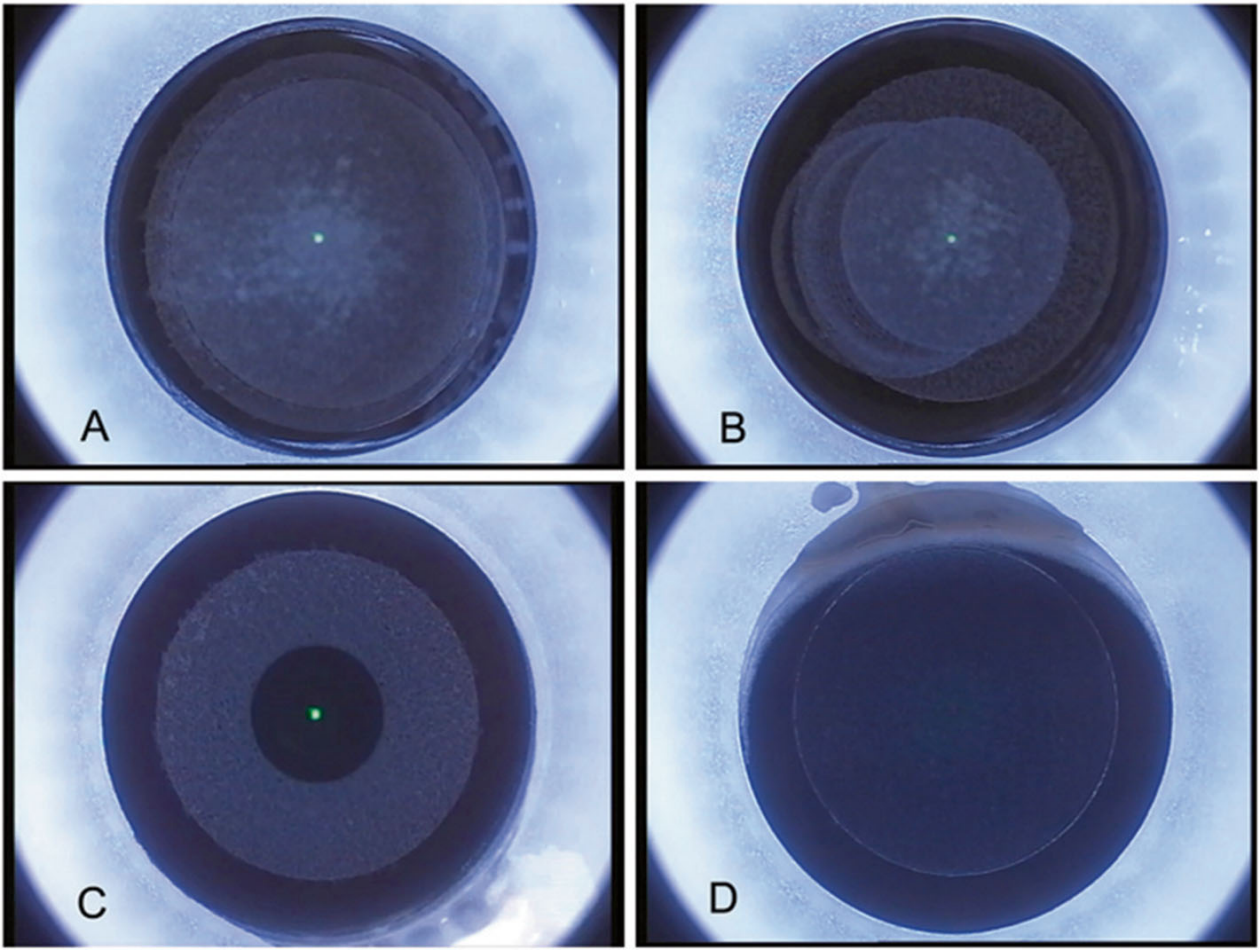
Images of suction loss during Small incision lenticule extraction surgery. **a**: Suction loss caused by a sudden head movement during the cap cutting procedure. **b**: Suction loss caused by eye rotation during cap cutting interface. **c**: Suction loss caused by large secretion of tears at the lenticule interface. **d:** Suction loss caused by conjunctive intrusion after lenticule side-cut was completed

In this study, suction loss was caused by several events. One was head movement, which could cause sudden suction loss without any sliding of the laser spot during laser cutting (Fig. 1a). Another was eye movement or rotation; compared with the suction loss caused by head movement, that caused by eye movement or rotation was often accompanied by the sliding of the laser cutting zone (Fig. 1b). Sudden eyelid squeezing or large amounts of tear secretion also caused suction loss, which was accompanied by a cascade of conjunctival intrusion (Fig. 1c and d).

Table 2 lists the number of suction loss cases due to head and eye movements. Head movements: Suction loss was caused by head movements in 16 (51.6%) eyes. During the procedure, the patients were instructed to relax and not to move their heads. However, anxious patients may be unable to comply and may remain tense. Muscle tension can result in natural protective mechanisms, including pulling or turning their heads away and raising or lowering their chin. Some patients may have tried to remain still, but their heads were slowly deviating to one side. Eye movements: Suction loss was caused by eye movements in 15 eyes (48. 4%). Once docking was completed, the patients were instructed to keep both eyes open during the entire laser cutting procedure and not to follow or search for the green light when it moves, blurs, or disappears. However, anxious patients could not follow the instructions or could not cooperate with the surgeon. Some patients would squeeze and blink or rotate their eyes abruptly. There were three common eye movements. The first was the Bell’s reflex, which is an involuntary upward shift of the eye. The second was following the green light. The third was sudden eye rotation.

**Table 2:** Causes of suction loss

Movement type		Suction loss (eye)	Incidence (%)
Head movement	Raise the chin	10	32.3
	Lower the chin	4	12.9
	Slight deviation of head position	2	6.4
Eye movement	Bell’s reflex	6	19.4
	Light tracking	4	12.9
	Eye rotation	5	16.1
Total suction loss		31	100

Table 3 shows the different categories of suction loss and the subsequent management strategies employed. According to the preprogrammed machine restart treatment module incorporated into the software of the VisuMax Femtosecond Laser, the laser cutting progress can be divided into five potential stages. Suction loss is categorized accordingly, and the recommended management varies between each category. Suction loss was experienced by 4 eyes in the lenticule stage (including lenticule interface and lenticule side cut) and by 27 eyes in the cap interface stage (including cap interface and small incision). Following the guideline of the software, we either restarted the procedure or changed to LASIK.

**Table 3:** Suction loss in various stages of femtosecond laser cutting progress and the subsequent management

Stage of suction loss		Suction loss (eye)	Incidence (%)	Subsequent management
Lenticule stage	lenticule interface (first 10%)	1	3.2	SMILE without any changes in the setting
	lenticule interface (10 to 100%)	3	9.7	Convert to femtosecond-assisted LASIK.
Cap stage	cap interface	19	61.3	SMILE without any changes in the setting
	small incision	8	25.8	SMILE can be restarted from the small incision
Total suction loss		31	100	

In 2014, the incidence of suction loss was 0 (0%) of 84 eyes. In 2015, the incidence was 11 (2.13%) of 516 eyes, the highest in the six consecutive years. It remained relatively low in the four subsequent years, 4 (0.34%) of 1163 eyes in 2016, 4 (0.24%) of 1653 eyes in 2017, 5 (0.22%) of 2264 eyes in 2018, and 7 (0.25%) of 2813 eyes in 2019.

## Discussion

Suction loss during SMILE surgery is a potential intraoperative complication, which might negatively influence the postoperative visual acuity. When suction loss occurs, management strategies include postponing surgery or immediate redocking [4, 13]. However, the drawbacks of immediate continuance using a redocking technique include increased patient anxiety, difficulties in observing the pupil center due to the presence of air bubbles, risks for treatment decentration due to difficulties in pinpointing, risks for uneven lamellar cuts [8], and increased anxiety of the surgeon. Therefore, correct and accurate responses based on guidelines and experience are important for good prognosis of visual acuity after suction loss.

After reviewing all cases of suction loss in our SMILE database, we found several factors that lead to suction loss, including head and eye movements during laser cutting, which can be associated with the tension and anxiety of the patients. Therefore, during preoperative education, we should not only instruct patients on how to gaze, but also emphasize the importance of not moving their heads and eyes. The correct placement of the body and the head is crucial. Thus, in addition to adjusting the headrest, soft pads may be placed under the patient’s head when necessary. In general, we must maintain the patient’s head at a horizontal level, which will help the patient to remain still during surgery and to refrain from raising or lowering their chin.

During the waiting period before the surgery, we observed some body language signals that might predict possible intraoperative suction loss. Some patients showed signs of anxiety, such as fidgeting, looking around, and talking endlessly, especially among the male patients. Our study shows that male patients (76.7%) had a significantly higher incidence of suction loss than female patients (23.3%). We should pay more attention on providing the necessary comfort and guidance to anxious patients as they may fail to follow the doctor’s instructions and to track the fixation light with their eyes, and they might make small oscillatory movements due to the enhanced arterial pulse of their upper body and head. In addition, we observed that suction loss occurs more frequently during the cap cutting periods. In our study, suction loss in 19 (61.3%) eyes occurred during the cap interface stage and 8 (25.8%) during the creation of small incision. The respective numbers were 45 and 15% as reported by Reinstein [6] and 51 and 3% as reported by Liu [13]. One possible explanation for this phenomenon is that the air bubbles generated from lenticule cutting obscure the patient’s vision. The patient loses sight of the green fixation light and panics, hence the movement.

Other risk factors, including the lack of surgical experience, increase the incidence of suction loss. The reported incidence of suction loss varies among studies. A review by Reinstein reported that the average global incidence of suction loss during SMILE surgery is 0.72% [6]. In studies involving large population of more than 1000 cases, the reported incidence of suction loss ranges from 0.17 to 2.10% [3, 6, 8, 10, 11, 13, 14]. As doctors become more experienced, the incidence of suction loss decreases. In our study, the incidence of suction loss in the six consecutive years were 0, 2.13, 0.34, 0.24, 0.22, and 0.25%, with an average incidence of 0.37%. The incidence was highest in 2015, during which the number of SMILE surgery performed in our facility rapidly increased. In the four subsequent years, the incidence of suction loss remained relatively low as the surgeon gained more experience. This phenomenon also reflects the learning curve of the surgeons (experience in surgical skills as well as communication techniques during the procedure). Osman reported that the incidence of suction loss decreased with surgical experience. In their first year, the incidence was 5.06%, whereas in their fifth year the number dropped to 1.84% [11]. However, the increase in surgical experience did not eliminate suction loss completely, which could not be fully explained by their learning curve, because suction loss often related to patient anxiety or movement [8].

According to Osman [11], some risk factors, such as a larger cap diameter and higher cylinder, might be related to a weaker grasp on the cornea by the suction cone or to a lower shape compatibility between the cornea and the cone. However, CCT, residual stromal thickness, cap thickness, and optical zone were not identified as important risk factors for suction loss. In other studies, smaller palpebral fissures, steep corneas, smaller corneal diameter, and conjunctival chemosis were identified as risk factors [13]. In our study, we observed that the first operative eye and male were important risk factors for suction loss, and this finding was supported by the result of a binary logistic regression analysis. The incidence of suction loss in the right eye (the first operative eye) is 23 (74.2%) eyes, consistent with Liu’s finding (77%) [13]. Thus, we should enhance preoperative education, especially among male patients.

This study stresses the importance of patient head stability in addition to controlling eye movement during the procedure. Surgeons should focus not only on the eyes, but also on the patient’s anxiety and body language.

This study has several limitations. First, the retrospective comparative case control design is at higher risk of selection bias. However, this design is more suitable to reflect the learning curve of a surgeon. Secondly, we have hypothesized that patient anxiety is a possible risk factor for suction loss, without statistical support. Future studies will assess the preoperative, intraoperative and postoperative patient anxiety levels. Finally, the number of patients who experienced suction loss was small. A larger sample would provide a better insight into the risk factor and causes of suction loss.

## Conclusions

The risk factors of suction loss were first operative eye and male sex. Head and eye movements due to patient anxiety are the most common direct causes of suction loss. Surgeon’s experience may help to reduce the incidence of suction loss. Surgeons should pay attention to patient education before surgery, to reinforcing communication and providing comfort during the procedure.

## Data Availability

The data have not been placed in any online data storage. The datasets generated and analyzed during the study are available upon request from the first author.

## Abbreviations

LASIK: Laser-assisted in situ keratomileusis
FS-LASIK: Femtosecond laser-assisted LASIK
SMILE: Small incision lenticule extraction
IOP: Intraocular ocular pressure
CCT: Central corneal thickness
OD: Right eye
OS: Left eye
SE: Spherical equivalent
RST: Residual stromal thickness
